# Supplementary figures and images for: AR-V7 exhibits non-canonical mechanisms of nuclear import and chromatin engagement in castrate-resistant prostate cancer
Source: eLife. 2022 Jul 18;11:e73396. doi: 10.7554/eLife.73396 (PMC9398446; doi:10.7554/eLife.73396)

Figure 6-source data 1

G

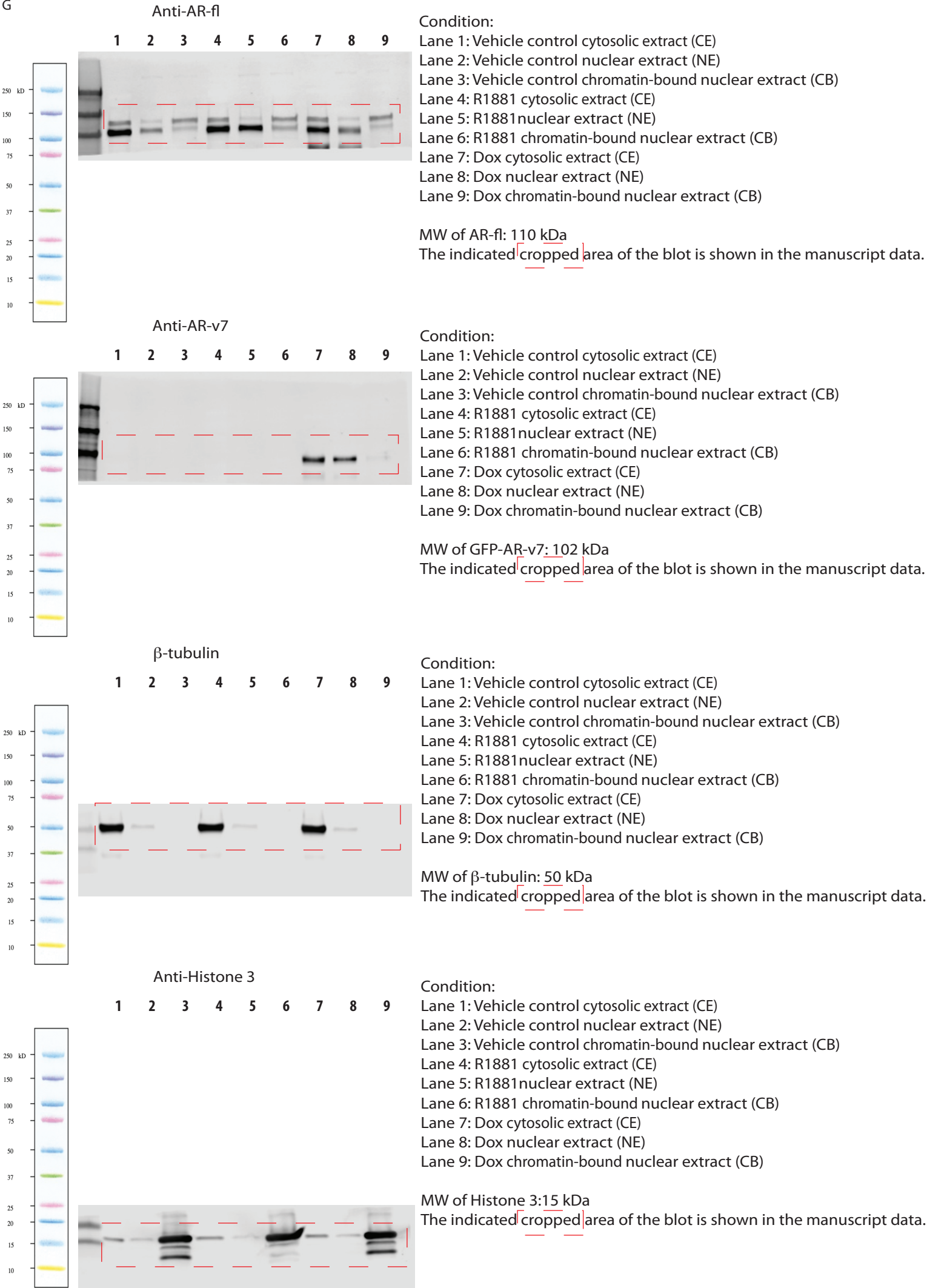

Supplement: Figure 6—source data 1. [file elife-73396-fig6-data1.pdf]
